# Urinary Metabolic Profiling During Epileptogenesis in Rat Model of Lithium–Pilocarpine-Induced Temporal Lobe Epilepsy

**DOI:** 10.3390/biomedicines13030588

**Published:** 2025-02-27

**Authors:** Fatma Merve Antmen, Emir Matpan, Ekin Dongel Dayanc, Eylem Ozge Savas, Yunus Eken, Dilan Acar, Alara Ak, Begum Ozefe, Damla Sakar, Ufuk Canozer, Sehla Nurefsan Sancak, Ozkan Ozdemir, Osman Ugur Sezerman, Ahmet Tarık Baykal, Mustafa Serteser, Guldal Suyen

**Affiliations:** 1 Department of Physiology, Graduate School of Health Sciences, Acibadem Mehmet Ali Aydinlar University, Istanbul 34752, Türkiye; merve.antmen@acibadem.edu.tr (F.M.A.);; 2 Biobank Unit, Acibadem Mehmet Ali Aydinlar University, Istanbul 34752, Türkiye; 3 Department of Medical Biochemistry, School of Medicine, Acibadem Mehmet Ali Aydinlar University, Istanbul 34752, Türkiye; 4 Medical Laboratory Techniques, Vocational School of Health Services, Acibadem Mehmet Ali Aydinlar University, Istanbul 34752, Türkiye; 5 Department of Molecular Biology and Genetics, Faculty of Arts and Sciences, Acibadem Mehmet Ali Aydinlar University, Istanbul 34752, Türkiye; 6 Department of Molecular Biology and Genetics, Inonu University, Malatya 44280, Türkiye; 7 School of Medicine, Acibadem Mehmet Ali Aydinlar University, Istanbul 34752, Türkiye; 8 Medical Biology, Department of Basic Medical Sciences, Acibadem Mehmet Ali Aydinlar University, Istanbul 34752, Türkiye; 9 Biostatistics and Medical Informatics, Department of Basic Medical Sciences, School of Medicine, Acibadem Mehmet Ali Aydinlar University, Istanbul 34752, Türkiye; 10 Acibadem Labmed Clinical Laboratories, Istanbul 34752, Türkiye; 11 Department of Physiology, School of Medicine, Acibadem Mehmet Ali Aydinlar University, Istanbul 34752, Türkiye

**Keywords:** epilepsy, urine, nuclear magnetic resonance, metabolomics, epileptogenesis, rat

## Abstract

**Background/Objectives**: Temporal lobe epilepsy (TLE) often develops following an initial brain injury, where specific triggers lead to epileptogenesis—a process transforming a healthy brain into one prone to spontaneous, recurrent seizures. Although electroencephalography (EEG) remains the primary diagnostic tool for epilepsy, it cannot predict the risk of epilepsy after brain injury. This limitation highlights the need for biomarkers, particularly those measurable in peripheral samples, to assess epilepsy risk. This study investigated urinary metabolites in a rat model of TLE to identify biomarkers that track epileptogenesis progression across the acute, latent, and chronic phases and elucidate the underlying mechanisms. **Methods**: Status epilepticus (SE) was induced in rats using repeated intraperitoneal injections of lithium chloride–pilocarpine hydrochloride. Urine samples were collected 48 h, 1 week, and 6 weeks after SE induction. Nuclear magnetic resonance spectrometry was used for metabolomic analysis, and statistical evaluations were performed using MetaboAnalyst 6.0. Differences between epileptic and control groups were represented using the orthogonal partial least squares discriminant analysis (OPLS-DA) model. Volcano plot analysis identified key metabolic changes, applying a fold-change threshold of 1.5 and a *p*-value < 0.05. **Results**: The acute phase exhibited elevated levels of acetic acid, dihydrothymine, thymol, and trimethylamine, whereas glycolysis and tricarboxylic acid cycle metabolites, including pyruvic and citric acids, were reduced. Both the acute and latent phases showed decreased theobromine, taurine, and allantoin levels, with elevated 1-methylhistidine in the latent phase. The chronic phase exhibited reductions in pimelic acid, tiglylglycine, D-lactose, and xanthurenic acid levels. **Conclusions**: These findings highlight stage-specific urinary metabolic changes in TLE, suggesting distinct metabolites as biomarkers for epileptogenesis and offering insights into the mechanisms underlying SE progression.

## 1. Introduction

Temporal lobe epilepsy (TLE) often occurs following initial brain damage, such as trauma or stroke, which triggers epileptogenesis—a process that transforms a healthy brain into one that is prone to spontaneous recurrent seizures (SRSs). Epileptogenesis progresses through three phases: (1) the acute phase, which occurs immediately after injury and is characterized by molecular and cellular responses; (2) the latent phase that is marked by ongoing molecular and structural changes without observable seizures; and (3) the chronic phase, during which SRSs emerge, driven by persistent epileptogenic changes [1,2,3,4]. Despite the importance of understanding these stages, no reliable biomarker can distinguish or monitor each phase. Electroencephalography (EEG), the standard diagnostic tool for epilepsy, cannot accurately predict epilepsy development after brain injury [5,6]. The current treatments for TLE rely heavily on anti-seizure medications, which primarily suppress seizure activity without addressing underlying neuropathological changes. These treatments often result in severe side effects and are ineffective in patients with drug-resistant TLE [7,8,9]. This highlights the critical need for phase-specific biomarkers to monitor TLE progression and facilitate the development of novel, targeted therapies.

Experimental animal models mimicking TLE are crucial for understanding epileptogenesis, improving current treatments, and developing novel therapeutic strategies. In experimental TLE models, epileptic seizures typically occur following status epilepticus (SE) induced by chemoconvulsants such as kainic acid pilocarpine. This is followed by a latent period lasting days to weeks, ultimately leading to SRSs. Among these models, the lithium–pilocarpine-induced SE model is one of the most widely used in research [10,11].

The exploration of urinary biomarkers for epilepsy is an emerging area of research that holds promise for improving the diagnosis, monitoring, and understanding of the pathophysiology of this complex neurological disorder. One of the primary advantages of urinary biomarkers is their non-invasive nature, which allows for easier patient compliance and repeated sampling. The exploration of urinary biomarkers in the context of neurological disorders has gained significant attention due to their potential for early diagnosis, disease progression monitoring, and treatment response evaluation [12].

The integration of advanced technologies such as proteomics and metabolomics has enhanced the discovery of novel urinary biomarkers. Metabolites are small molecules produced during metabolic processes, and their profiles can vary significantly between healthy and diseased states. Therefore, they are crucial biomarkers. In epilepsy, for example, pyridoxal phosphate and its derivatives are notable biomarkers associated with neurotransmitter metabolism. Pyridoxine-dependent epilepsy, a rare metabolic disorder caused by mutations in the *ALDH7A1* gene, is characterized by elevated urinary levels of α-aminoadipic semialdehyde (α-AASA), a key diagnostic and monitoring biomarker [13,14]. This condition highlights the role of metabolites in understanding disease mechanisms and guiding effective treatments, such as pyridoxine therapy [14].

Nuclear magnetic resonance (NMR) spectroscopy is a highly informative technique that uses the magnetic properties of atomic nuclei to non-destructively analyze the structure, identity, concentration, and behavior of molecules in solid or liquid samples. The key advantages of this method include being non-destructive and non-invasive, the ease of sample preparation, and the high reproducibility of the results [15,16]. Metabolomic studies using NMR spectroscopy in patients with epilepsy and animal models are rare [17]. Although blood and brain samples are commonly analyzed, urine—a readily accessible biofluid with strong relevance to CNS disorders—offers significant potential for metabolic research. Investigating urinary metabolites during different epilepsy phases could address gaps in the literature and provide deeper insights into metabolic changes throughout disease progression. Therefore, this study examined the alterations in urine metabolic profiles via NMR spectroscopy and the associated metabolic pathways in detail during the three principal epileptogenesis periods in a lithium–pilocarpine-induced TLE rat model, which is one of the most representative models of TLE. This study aimed to identify potential biomarkers of epileptogenesis.

## 2. Materials and Methods

### 2.1. Animals

In this study, 34 young male Sprague Dawley rats weighing 250–450 g were used. Of these, 30 rats were obtained from the Acıbadem University Experimental Animal Research Center. The rats were acclimated to the laboratory environment for 7 days before the experiments. To minimize external stress factors and facilitate adaptation, a handling protocol was implemented, in which each rat was gently held by hand for 5 min daily over 5 consecutive days. The rats were housed under controlled conditions at room temperature (24 °C ± 1 °C) with a 12 h light–dark cycle (lights on at 7:00 a.m.) and were provided with ad libitum access to food and water. Every effort was made to minimize animal suffering and reduce the number of animals used. Considering the challenging, prolonged, and complex nature of epilepsy studies, difficulties were encountered in ensuring adequate food and water intake after status epilepticus (SE) induction. These challenges were addressed by providing supportive measures such as wetting food and placing it in cages to facilitate its consumption. To prevent irritation or injury, each rat subjected to SE was housed individually throughout the process. All procedures associated with animal care, experimental protocols, and euthanasia were approved by the Experimental Animal Research Ethics Committee of Acıbadem University (approval number: HDK-2022/80).

### 2.2. Chemicals

Lithium chloride, methylscopolamine, and pilocarpine hydrochloride were obtained from Sigma-Aldrich^®^ (St. Louis, MO, USA), and thiopental sodium was obtained from Ibrahim Etem, a member of the Menarini Group in Türkiye. For intraperitoneal administration, lithium chloride was dissolved in injectable water at a dose of 3 mEq/kg. Methylscopolamine (1 mg/kg) and pilocarpine hydrochloride (20 mg/kg) were prepared in 0.9% saline solution and administered intraperitoneally. Similarly, thiopental sodium (30 mg/kg) was dissolved in injectable water and administered via the same route.

### 2.3. Epileptogenesis Induction and Group Allocation

In this study, SE was induced, and a rat model of TLE was established through repeated intraperitoneal injections of low-dose lithium chloride and pilocarpine hydrochloride [11]. Lithium chloride (3 mEq/kg; intraperitoneally) was administered initially, followed by pilocarpine hydrochloride (20 mg/kg; intraperitoneally) after 20 h. Additional doses of pilocarpine hydrochloride (20 mg/kg; intraperitoneally) were administered at 30 min intervals until SE was achieved, with a maximum of five doses. To counteract peripheral cholinergic effects, methylscopolamine (1 mg/kg; intraperitoneally) was injected 30 min before the first pilocarpine hydrochloride dose. SE was evaluated behaviorally using a modified Racine scale (1972), as adapted by Wamil et al. (1989) [18,19]. To minimize mortality, thiopental sodium (30 mg/kg; intraperitoneally) was administered 90 min after SE onset, with up to two additional doses administered at 10 min intervals if required.

The rats were randomly divided into six groups, and this study was completed with the following experimental groups:

(1) TLE acute phase group (SE-48h, *n* = 5): SE was induced, and urine samples were collected during the acute phase of epileptogenesis.
(2) Control acute phase group (C-48h, *n* = 3): SE was not induced; however, urine samples were collected during the time corresponding to the acute phase in the epilepsy group.
(3) TLE latent phase group (SE-1wk, *n* = 5): SE was induced, and urine samples were collected during the latent phase of epileptogenesis.
(4) Control latent phase group (C-1wk, *n* = 7): SE was not induced; however, urine samples were collected during the time corresponding to the latent phase in the epilepsy group.
(5) TLE chronic phase group (SE-6wk, *n* = 8): SE was induced, and urine samples were collected during the chronic phase of epileptogenesis.
(6) Control chronic phase group (C-6wk, *n* = 6): SE was not induced; however, urine samples were collected during the time corresponding to the chronic phase in the epilepsy group.

### 2.4. Urine Sample Collection

This study identified metabolic alterations in the urine of rats following the induction of epileptogenesis. Thus, the three time points of epileptogenesis were analyzed. The time points reflecting the different phases of epileptogenesis were selected based on a literature review [6,20,21]. Urine samples corresponding to the specified phases of epileptogenesis were collected using metabolic cages. Each rat was housed in a metabolic cage for 24 h at the time point corresponding to the target phase of epileptogenesis, and 24 h urine samples were collected [22]. The collected urine samples were centrifuged at 12,000 rpm for 5 min at 4 °C. The supernatants were then rapidly fresh-frozen in liquid nitrogen and stored at −80 °C for further analysis [23], as detailed below.

### 2.5. Preparation of Samples for NMR Spectrometry and NMR Process

Before NMR analysis, urine samples stored at −80 °C were thawed at 4 °C. Following a brief vortexing step, the samples were centrifuged at 14,000× *g* for 5 min at 4 °C, and the supernatants were separated. The obtained supernatants (900 µL) were mixed with a 100 µL volume of buffer solution (purchased directly from Bruker) in a microcentrifuge tube. This mixture (1000 µL) was then transferred to 5 mm SampleJet NMR tubes for further analysis [24,25].

Spectroscopic analyses were performed using a Bruker Avance III HD series spectrometer with an operating frequency of 600 MHz. The system featured a 5 mm broadband inverse probe and was further optimized using a Bruker SampleJet robotic system for sample cooling. The system was maintained at a constant temperature of 5 °C. The analyses began following a meticulous calibration process, as previously described [26].

The “Electronic Reference for In Vivo Concentration determination” method was used to generate reference standards [25]. To enable automated annotation and quantification of disease-associated small metabolites, ^1^H NOESY spectra were recorded. The resulting dataset was prepared using the B.I.QUANT-PS™ method (B.I.: Bruker BioSpin GmbH, Ettlingen, Germany), which comprises 150 urine metabolites [24,25,27].

### 2.6. Statistical Analysis

Metabolite statistical analysis was conducted using MetaboAnalyst 6.0 (www.metaboanalyst.ca, accessed multiple times between January and February 2025) [28]. Before ^1^H-NMR-based metabolomics data analysis, the dataset was subjected to filtering and integrity checks to ensure the completeness and accuracy of the essential information. This included categorizing the data, verifying non-negative values for compound concentrations or peak intensities, and addressing any missing entries. Normalization was performed using the MetaboAnalyst 6.0 normalization module, with logarithmic transformation (base 10) and automatic scaling applied to standardize the data.

To investigate differences between the TLE model groups and their corresponding controls, multivariate analysis was performed using the orthogonal partial least squares discriminant analysis (OPLS-DA) model [29]. The permutation test was carried out to validate the OPLS-DA model. Univariate analysis was also performed as part of the exploratory data analysis. A significance threshold of *p* ≤ 0.05 was applied. Fold-change (FC) analysis with a cutoff of 1.5 was performed to identify metabolites potentially associated with epileptogenesis, as previously described [6]. Moreover, volcano plot analysis was performed to highlight key features based on both statistical and biological significance, using an FC threshold of 1.5 and a *t*-test significance level of *p* ≤ 0.05. Metabolites were considered significantly different if the FC ≥ 1.5 and *p* ≤ 0.05, unless stated otherwise. For metabolic pathway analysis, MetaboAnalyst 6.0 and the KEGG metabolic pathway database for *Rattus norvegicus* were used. Furthermore, MetaboAnalyst 6.0 was used for the enrichment analysis.

## 3. Results

### 3.1. Total Profile of the Metabolic Changes in Urine

**Figure 1:**
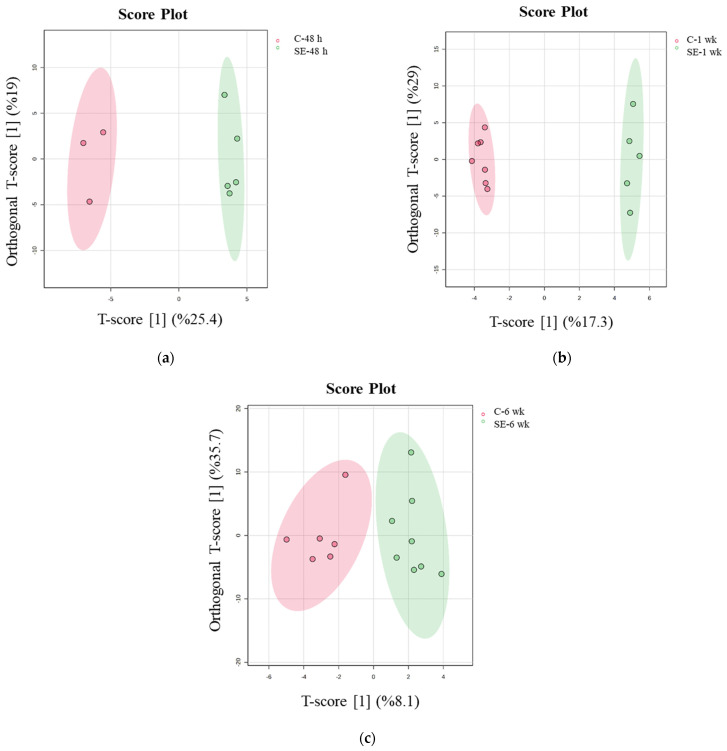
Score plots of the two-component OPLS-DA model for urine samples show the differentiation at three time points of epileptogenesis, (**a**) C−48 h vs. SE−48 h, (**b**) C−1 wk vs. SE−1 wk, and (**c**) C−6wk vs. SE−6 wk, based on NMR data.

The OPLS-DA model based on urine samples revealed significant differences between the three phases of epileptogenesis: between the SE-48h and C-48h groups (orthogonal T-score = 19%, T-score = 25.4%) (Figure 1a), the SE-1wk and C-1wk groups (orthogonal T-score = 29%, T-score = 17.3%) (Figure 1b), and the SE-6wk and C-6wk groups (orthogonal T-score = 35.7%, T-score = 8.1%) (Figure 1c).

The OPLS-DA model exhibited strong predictive power in the acute phase (Q^2^ = 0.843, *p* = 0.01), moderate performance in the latent phase (Q^2^ = 0.499, *p* = 0.06), and no significant predictive power in the chronic phase (Q^2^ = −0.0335, *p* = 0.36), as shown in Figure S1. This is likely due to metabolic stabilization, increased inter-individual variability, and long-term adaptations to chronic epilepsy. To address this limitation, we performed additional univariate analyses and pathway-based interpretations to further investigate potential long-term metabolic changes.

### 3.2. Changes in Specific Metabolites in Urine

Metabolites play a crucial role in understanding the complexity of biochemical processes, providing insights into both health and disease states. To investigate potential changes in the urinary metabolic profile during epileptogenesis, comparisons were made between the SE and control groups. Thus, the dataset was subjected to univariate analysis. Student’s *t*-test and fold-change (FC) analyses (*p* ≤ 0.05 and FC ≥ 1.5) were performed to identify significant differences in metabolic features between the SE and control groups.

**Figure 2:**
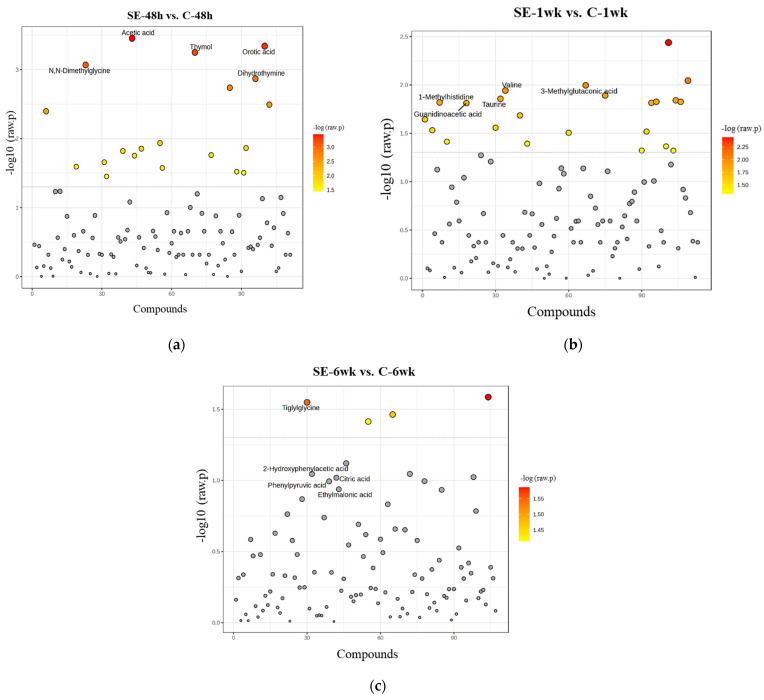
Significantly altered metabolites in urine samples. Statistical results of *t*-tests for (**a**) C−48 h vs. SE−48 h, (**b**) C−1 wk vs. SE−1 wk, and (**c**) C−6wk vs. SE−6 wk based on NMR data. The scatter plot displays the distribution of metabolites based on their -log10(*p*-value). Colored dots indicate metabolites with relatively lower *p*-values, with a gradient from yellow to red representing increasing statistical significance, while grey colored represent unsignificant ones.

In the SE-48h group, compared to the C-48h group, statistically significant decreases were observed in the concentrations of the following metabolites: 1-methylhydantoin, 2-oxoglutaric acid, adenine, allantoin, citric acid, fumaric acid, hippuric acid, L-pyroglutamic acid, N,N-dimethylglycine, orotic acid, pyruvic acid, tartaric acid, taurine, theobromine, tiglyglycine, and trigonelline. Conversely, the concentrations of acetic acid, dihydrothymine, thymol, and trimethylamine (TMA) were significantly increased in the SE-48h group compared to the C-48h group (Figure 2a). In the SE-1wk group, which represents the latent phase of epileptogenesis, a significant increase in the 1-methylhistidine (1-MH) concentration was observed compared with that in the C-1wk group. However, significant decreases in the concentrations of neopterin, D-glucose, 3-methylglutaconic acid, valine, glycolic acid, taurine, theobromine, caffeine, uracil, allantoin, guanidinoacetic acid, hippuric acid, creatinine, proline betaine, 1-methylguanidine, 4-pyridoxic acid, pantothenic acid, alanine, syringic acid, inosine, 1-methylhydantoin, and oxipurinol were observed compared with those in the C-1wk group (Figure 2b). In the SE-6wk group, which corresponds to the chronic phase, significant decreases in the concentrations of D-lactose, pimelic acid, tiglyglycine, and xanthurenic acid were observed compared with those in the C-6wk group. However, no metabolites exhibited significant increases in the SE-6wk group (Figure 2c).

FC analysis with a threshold of 1.5 was performed to pinpoint metabolites potentially associated with epileptogenesis. Table 1, Table 2 and Table 3 present metabolites with FC values ≥ 1.5, corresponding to the following time points: 48 h, 1 week, and 6 weeks after SE induction, respectively.

**Figure 3:**
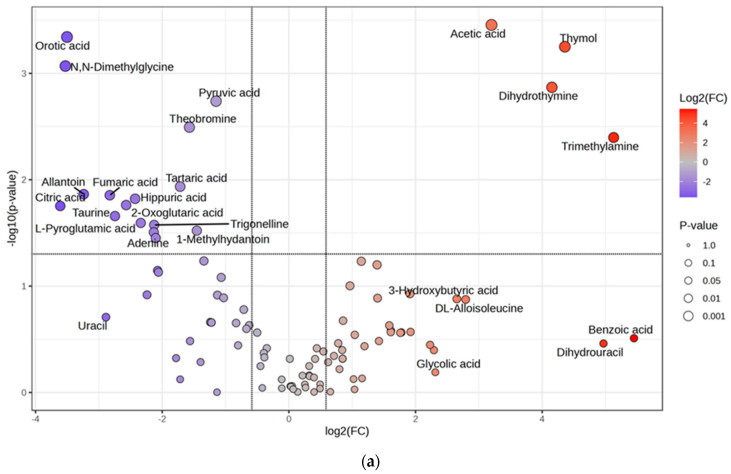

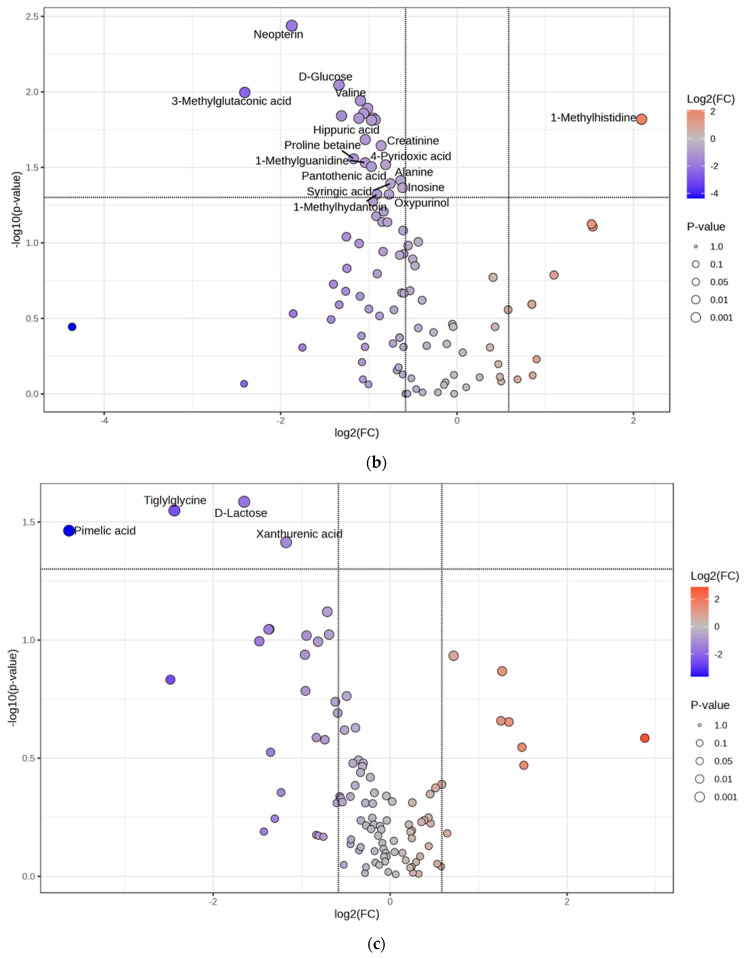
Volcano plot analysis of (**a**) C−48 h vs. SE−48 h, (**b**) C−1 wk vs. SE−1 wk, and (**c**) C−6wk vs. SE−6 wk based on NMR urinary metabolite data. The color gradient represents log2(FC), where red indicates increased metabolites, blue indicates decreased metabolites, while grey points represent non-significant metabolites. The size of the points corresponds to statistical significance (*p*-value), with larger points indicating higher significance.

In addition to the aforementioned findings, volcano plot analysis (Figure 3) revealed significant changes in the levels of metabolites across different phases of epileptogenesis (Table 4). In the SE-48h group, most metabolites exhibited a decreasing trend, with significant reductions in compounds such as orotic acid, pyruvic acid, and citric acid, whereas acetic acid, thymol, dihydrothymine, and TMA increased. During the latent phase, metabolites such as taurine, D-glucose, and caffeine decreased; however, no metabolites significantly increased. Similarly, in the chronic phase, compounds such as tiglylglycine and pimelic acid decreased; however, no metabolites increased. Overall, the most substantial changes occurred at 48 h and 1 week after SE induction, with notable increases detected only at 48 h.

### 3.3. Metabolic Pathway Analysis of Urinary Metabolites

Metabolic pathway analysis was performed using MetaboAnalyst 6.0 and the KEGG metabolic pathway database. Table 5 presents the results of the analysis. This table lists the significantly altered metabolites (*p* ≤ 0.05 and FC ≥ 1.5) and presents the pathways that exhibited statistical significance at the *p* ≤ 0.05 level. This study revealed significant associations between metabolites and pathways during the acute and latent phases of the experiment. However, no significant metabolite-based pathway effects were observed during the chronic phase of the experiment.

#### 3.3.1. Alanine, Aspartate, and Glutamate Metabolism

In the acute phase, significant changes were observed in the levels of citric acid, pyruvic acid, fumaric acid, and 2-oxoglutaric acid. Pathway analysis revealed that “alanine, aspartate, and glutamate metabolism” was statistically significant. All four metabolites are part of this pathway. No significant changes in this pathway were observed during the latent or chronic phases.

#### 3.3.2. Glyoxylate and Dicarboxylate Metabolism

During the acute phase, significant changes in citric acid, pyruvic acid, and acetic acid levels were detected. Pathway analysis indicated that the “glyoxylate and dicarboxylate metabolism” pathway was statistically significant, which is in agreement with the observed changes in these metabolites. No significant changes in this pathway were observed during the latent or chronic phases.

#### 3.3.3. Glycine, Serine, and Threonine Metabolism

Consistent with plasma findings, the analysis of urinary metabolites identified “glycine, serine, and threonine metabolism” as significant during the acute phase. Changes in metabolites such as N,N-dimethylglycine and pyruvic acid were associated with this pathway. No significant changes in this pathway were observed during the latent or chronic phases.

#### 3.3.4. TCA Cycle

Pathway analysis revealed significant alterations in the TCA cycle between the SE-48h and C-48h groups. Metabolites such as 2-oxoglutaric acid, citric acid, pyruvic acid, and fumaric acid were significantly altered (*p* ≤ 0.05 and FC ≥ 1.5). No significant changes in the TCA cycle were observed during the latent or chronic phases.

#### 3.3.5. Pyruvate Metabolism

Pathway analysis identified significant changes in the “pyruvate metabolism” pathway between the SE-48h and C-48h groups, with notable differences in the pyruvic acid, fumaric acid, and acetic acid levels. No significant findings for this pathway were observed during the latent or chronic phases.

#### 3.3.6. Glycolysis/Gluconeogenesis

The “glycolysis/gluconeogenesis” pathway showed statistically significant changes in the acute phase, associated with pyruvic acid and acetic acid levels, and in the latent phase, which was associated with D-glucose levels. No significant changes were found or observed in this pathway during the chronic phase.

#### 3.3.7. Pyrimidine Metabolism

In the acute phase, the “pyrimidine metabolism” pathway showed significant changes, primarily driven by alterations in orotic acid concentrations (*p* ≤ 0.05 and FC ≥ 1.5). No significant changes in this pathway were observed during the latent or chronic phases.

#### 3.3.8. Pentose Phosphate Pathway

The “pentose phosphate pathway” showed statistically significant changes only during the latent phase, driven by alterations in D-glucose levels (*p* ≤ 0.05 and FC ≥ 1.5).

#### 3.3.9. Caffeine Metabolism

Statistically significant changes in “caffeine metabolism” were observed during both the acute and latent phases. Theobromine levels decreased in both phases, while caffeine levels exhibited a significant decrease only in the latent phase. No significant findings were observed in the chronic phase.

#### 3.3.10. Taurine and Hypotaurine Metabolism

The “taurine and hypotaurine metabolism” pathway was significantly altered during the acute and latent phases, with taurine showing a significant decrease in both phases (*p* ≤ 0.05 and FC ≥ 1.5). No significant changes were observed during the chronic phase.

#### 3.3.11. Purine Metabolism

The “purine metabolism” pathway exhibited significant changes during both the acute and latent phases. Allantoin levels significantly decreased in both phases, while inosine exhibited a significant decrease only in the latent phase. No significant changes were observed during the chronic phase.

#### 3.3.12. Selenocompound Metabolism

The “selenocompound metabolism” pathway showed exhibited significant changes only during the latent phase, primarily driven by alterations in alanine levels (*p* ≤ 0.05 and FC ≥ 1.5).

#### 3.3.13. Primary Bile Acid Biosynthesis

The “primary bile acid biosynthesis” pathway was significantly altered only during the latent phase, with taurine being the sole metabolite associated with this pathway (*p* ≤ 0.05 and FC ≥ 1.5). No significant changes were observed during the acute or chronic phases.

### 3.4. Enrichment Analysis of Urinary Metabolites

**Figure 4:**
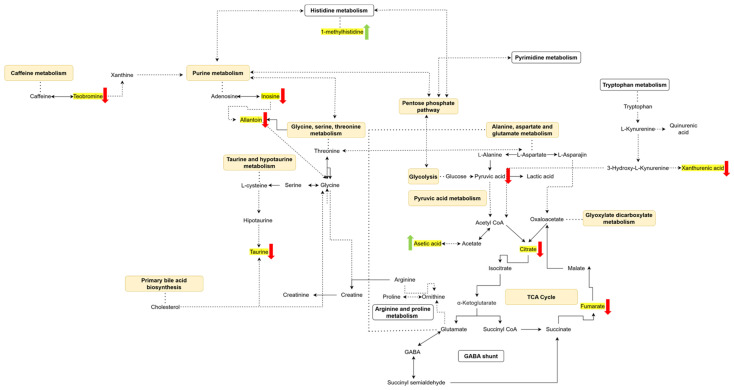
Key metabolic pathways and associated metabolites in epileptogenesis. Dashed arrows indicate multi-step transformations between two metabolites, while solid arrows represent single-step transformations. Double-headed arrows denote bidirectional transformations, and single-headed arrows indicate unidirectional transformations. Metabolites highlighted in yellow represent changes detected in urine. Yellow rectangles correspond to pathways affected exclusively in urine. Green arrows show metabolites that increased in urine, whereas red arrows indicate metabolites that decreased in urine.

Figure 4 presents the key metabolic pathways and associated metabolites identified through pathway analysis, which was conducted independently of the individual examination of plasma and urine metabolites at each stage of epileptogenesis. These pathways and metabolites met the criteria of *p* ≤ 0.05 and FC ≥ 1.5. In conclusion, various metabolic pathways and metabolites exhibited significant changes across the different phases of epileptogenesis.

**Figure 5:**
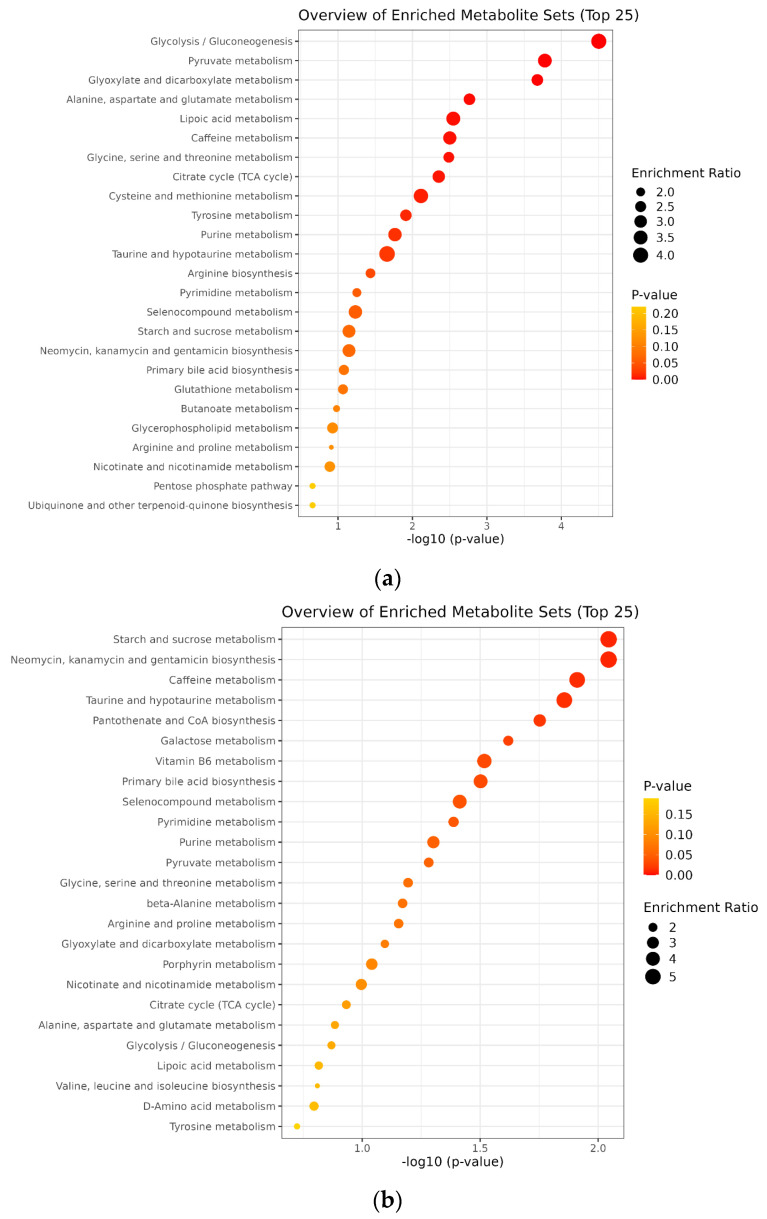

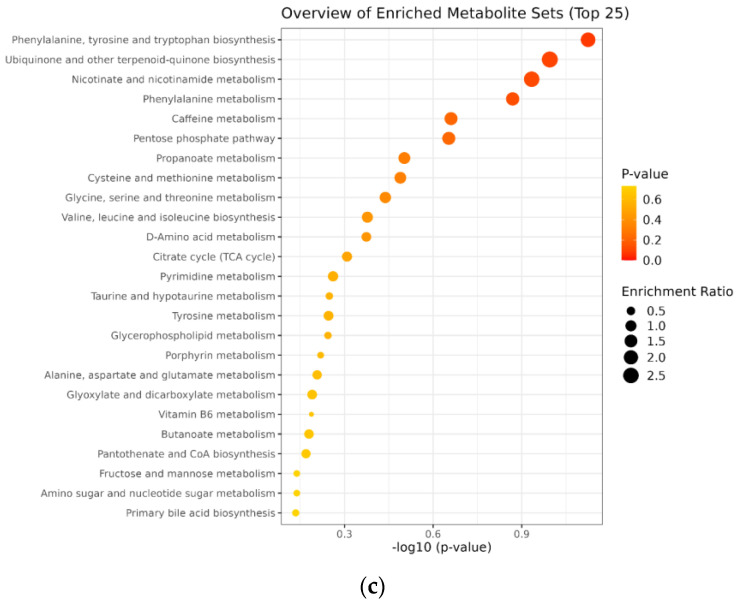
Enrichment analysis of significantly altered metabolic pathways in (**a**) acute phase, (**b**) latent phase, and (**c**) chronic phase of epileptogenesis. The top 25 enriched metabolic pathways are displayed based on pathway impact and statistical significance. The *x*-axis represents the −log10(*p*-value), indicating statistical significance, while the bubble size corresponds to the enrichment ratio, reflecting the degree of pathway involvement. The color intensity represents the *p*-value, with red indicating stronger significance.

Enrichment analysis revealed that glycolysis/gluconeogenesis; pyruvate metabolism; glyoxylate and dicarboxylate metabolism; alanine, aspartate, and glutamate metabolism; and the TCA cycle were among the most significantly altered pathways in the acute phase (Figure 5a). For the latent phase, pathways related to amino acid metabolism (e.g., cysteine and methionine metabolism, tyrosine metabolism, and taurine/hypotaurine metabolism) and lipid metabolism (e.g., glycerophospholipid metabolism, nicotinate and nicotinamide metabolism) also showed significant enrichment (Figure 5b). Pathway enrichment analysis identified key metabolic alterations, with the significant involvement of amino acid metabolism (e.g., phenylalanine, tyrosine, and tryptophan biosynthesis; glycine, serine, and threonine metabolism), energy metabolism (e.g., citrate cycle (TCA cycle), propanoate metabolism), and lipid metabolism (e.g., glycerophospholipid metabolism, nicotinate and nicotinamide metabolism). Several pathways related to neurotransmitter function and oxidative stress regulation, such as taurine and hypotaurine metabolism and cysteine and methionine metabolism, were also enriched.

## 4. Discussion

Central biomarkers provide valuable insights into brain structure and function but require advanced equipment, making them costly and impractical for frequent monitoring. Peripheral blood biomarkers are a less invasive option, but blood collection can still be inconvenient and unsuitable for continuous tracking. In contrast, reliable urinary biomarkers have the potential to transform epilepsy risk assessment by allowing for regular, non-invasive monitoring. They could help detect seizure risk early, track disease progression, and assess treatment effectiveness with minimal patient discomfort. As research progresses, incorporating urinary biomarkers into clinical practice could lead to personalized and timely interventions, ultimately enhancing patient care and outcomes. However, this area of research is still developing, and further studies are needed. In accordance with this objective, we conducted a comprehensive exploratory study encompassing stage-specific urine metabolic analysis during epileptogenesis.

Several biomarkers have been investigated for epilepsy risk assessment, encompassing both central and peripheral indicators [30,31]. Research on urinary biomarkers for epilepsy is still emerging. A study found volatile organic compounds in the urine of amygdala-kindled mice, indicating their potential as non-invasive epilepsy markers [32].

NMR-based metabolomics is a valuable tool for detecting metabolic changes in neurological disorders, aiding in early diagnosis and monitoring. In Alzheimer’s disease, studies on blood, CSF, urine, and saliva have identified disruptions in energy metabolism, oxidative stress, and neurotransmitter pathways [33]. Similarly, NMR-based urine analysis in autism models has revealed metabolic disturbances [34]. In epilepsy research, previous studies have mainly focused on acute seizure models or brain tissue analysis, with limited investigation into urinary metabolites [17]. The use of ^1^H-NMR spectroscopy to analyze a broad range of metabolites across different stages of epileptogenesis remains unexplored.

This study aims to investigate metabolic profile changes and associated metabolic pathways during the three main phases of epileptogenesis in a lithium–pilocarpine-induced TLE model, one of the most representative models of TLE [10,11]. Our study uniquely contributes by providing a comprehensive annotation and quantitation of diverse urinary metabolites across all three stages of epileptogenesis.

We identified potential biomarkers at each stage of epileptogenesis following SE induction. A highly innovative approach was employed, using the ^1^H-NMR technique, which is recognized as an effective method for analyzing molecular structures without damaging the collected samples. The results revealed distinct urinary metabolite alterations at different stages of epileptogenesis, underscoring the involvement of multiple metabolic pathways.

Pyruvate, derived from glucose via glycolysis, supports the TCA cycle through its conversion to acetyl-CoA under aerobic conditions [35]. Pyruvate’s neuroprotective benefits have been demonstrated in stroke, traumatic brain injury, and hypoglycemia [36,37,38]. Our study showed a significant decrease in urinary pyruvate levels during the acute phase of epileptogenesis in rats. This reduction may be associated with various metabolic and cellular responses during this phase. Epilepsy involves alterations in cellular energy metabolism, with seizures significantly increasing the ATP demand. The onset of epileptic seizures in the acute phase rapidly increases the energy demand in the brain tissue, resulting in the diversion of pyruvate to mitochondria for energy production, thereby reducing its excretion in the urine. Under hypoxic conditions, pyruvate is converted to lactate by lactate dehydrogenase (LDH) to facilitate anaerobic energy production. Increased LDH activity has been observed in some epilepsy kindling models [39]. Mitochondria play a critical role in intermediary metabolism and cellular energy regulation. Understanding mitochondrial dysfunction in epilepsy remains challenging, particularly in discerning whether mitochondrial oxidative stress is a cause or a consequence of seizures. Mitochondrial dysfunction, which is associated with conditions such as traumatic brain injury and seizures, highlights the critical role of pyruvate and lactate in the energy supply for neural cells [40].

Distinct metabolic differences were observed in urine during the acute phase of epileptogenesis, particularly reflected by disruptions in the TCA cycle. Consistent with our findings, impairments in the TCA cycle function have been reported in both the cerebral cortex and hippocampus of mice showing SRSs [41]. Our detailed analysis of the TCA cycle highlights the interaction between glycine, serine, and threonine metabolism and the TCA cycle as a key focus. The conversion of these amino acids to pyruvate plays a crucial role in supporting TCA cycle metabolites as anaplerotic substrates, which are vital for maintaining TCA cycle homeostasis [42]. This interaction highlights the metabolic flexibility and complex balance between the pathways required to optimize cellular function and energy production.

In the acute phase of epileptogenesis, the interplay between glycine, serine, and threonine metabolism and the TCA cycle, particularly evident in urinary metabolite changes, is significant for metabolic regulation. Notably, citric acid, a critical TCA cycle intermediate, exhibited a statistically significant decrease in urine during the acute phase, consistent with reports of reduced citrate levels in brain tissues in acute chemical kindling models [17]. This reduction may result from increased neuronal demand for intermediates such as citrate and 2-oxoglutarate, precursors for glutamate, during excessive neuronal excitation and glutamate release in epilepsy. Such demands may lead to their uptake from the circulation and reduced excretion in the urine. Alongside citric acid, fumaric acid and 2-oxoglutaric acid levels were also significantly reduced in urine during the acute phase, suggesting decreased aerobic respiration capacity, increased energy demand during seizures, local hypoxia, and mitochondrial dysfunction. These findings indicate a temporary reliance on anaerobic pathways for energy production in the brain cells. Therefore, the urinary levels of metabolites such as pyruvate, citric acid, 2-oxoglutaric acid, and fumaric acid could be potential biomarkers for identifying the acute phase of epileptogenesis. Tracking these metabolites over time may also help manage the progression of the condition.

In this study, increased acetic acid levels were observed in the urine samples obtained from rats during the acute phase of epileptogenesis. Consistent with our findings, elevated acetic acid levels were observed in brain tissue samples from patients with epilepsy compared with those in brain tissue samples from healthy controls and in the cerebrospinal fluid of dogs with idiopathic epilepsy [43,44]. During the acute phase following SE, increased urinary acetic acid levels may be associated with alterations in energy metabolism, lactic acidosis, and ketosis. The heightened energy demand during SE depletes glucose rapidly, causing a metabolic shift toward the use of fatty acids and proteins as energy sources. This shift increases the production of acidic metabolites, such as ketone bodies and acetic acid, which are detectable in urine. Furthermore, anaerobic glycolysis, driven by increased energy demand and muscle activity, contributes to lactic acidosis, prompting the body to excrete acidic metabolites such as acetic acid through urine. Furthermore, acetic acid produced by gut microbial fermentation, influenced by altered gut microbiota, may play a significant role in the pathophysiology of TLE [45]. These findings suggest that acetic acid can be a biomarker for the acute phase of epileptogenesis, warranting further research to deepen our understanding of the process.

Pathway-based analyses also revealed significant alterations in various metabolic pathways, particularly those involving alanine, aspartate, and glutamate metabolism; glyoxylate and dicarboxylate metabolism; glycine, serine, and threonine metabolism; pyruvate metabolism; and glycolysis/gluconeogenesis. These findings highlight the broad metabolic shifts associated with epileptogenesis.

Urinary glycine, serine, and threonine metabolism demonstrated an indirect relationship with purine metabolism. Pathway analyses revealed that inosine, uric acid, and allantoin are interconnected components of purine metabolism. Inosine can be catabolized into uric acid, which is further broken down into allantoin. In our study, allantoin levels decreased in both the acute and latent phases of epileptogenesis, whereas inosine levels decreased only in the latent phase. Uric acid functions as a potent antioxidant and reactive oxygen species (ROS) scavenger, and allantoin serves as a stable marker of oxidative stress independent of uric acid levels [46,47]. The low urinary allantoin levels during epileptogenesis may serve as an indicator of altered purine metabolism and compromised antioxidant defense. Allantoin can be a valuable biomarker for monitoring the epileptogenesis process and guiding therapeutic strategies. In contrast, inosine, a key metabolite in purine biosynthesis and degradation, also acts as a bioactive molecule regulating RNA editing, enzyme activity, and signaling pathways [48]. It has demonstrated neuroprotective and neuromodulatory effects in neurological disorders, including Parkinson’s disease, by increasing uric acid levels and scavenging ROS [49,50]. Furthermore, recent studies have indicated a mechanistic association between inosine and gut microbiota, suggesting that changes in the microbiota during epileptogenesis could influence inosine production [51,52]. Further investigations of purine-metabolism-related metabolites, such as allantoin and inosine, in animal models and clinical studies could provide deeper insights into epileptogenesis and antioxidant defense mechanisms.

Mitochondrial dysfunction also exacerbates neuroinflammation and oxidative stress, disrupting pyrimidine metabolism [53]. Orotic acid, a precursor in pyrimidine biosynthesis, is produced by dihydroorotate dehydrogenase in the mitochondria and is converted to uridine monophosphate in the cytoplasm [54]. Our findings revealed significantly reduced urinary orotic acid levels during the acute phase of epileptogenesis, likely reflecting the increased metabolic demand for DNA repair and cellular maintenance. Oxidative stress and heightened nucleotide consumption for mitochondrial DNA repair, such as mitochondrial base excision repair (mtBER) activation, may drive this reduction [55,56]. In the chronic phase, insufficient mtBER activity could contribute to mitochondrial instability and oxidative stress. Orotic acid’s role in gene regulation [57,58,59] and its acute-phase changes suggest its potential as a biomarker and therapeutic target in epileptogenesis.

This study identified the primary bile acid biosynthesis pathway as significant in the urinary analysis, with taurine as the only associated metabolite. Previous studies have reported inconsistent findings on taurine levels in epileptic brain tissue [60]. In this study, urinary taurine levels significantly decreased during both the acute and latent epileptogenesis phases. Similar reductions were observed in patients with untreated pediatric epilepsy, supporting its biomarker potential [61]. Taurine’s roles in neurotransmission, osmoregulation, antioxidant defense, and calcium regulation make it a neuroprotective and anticonvulsant candidate [60,62,63,64]. Its reduction in urine may reflect increased brain demand, renal reabsorption, or metabolic stress. Taurine’s biological importance highlights its promise as a biomarker and therapeutic target, with taurine derivatives offering potential in epilepsy treatment [65,66].

Theobromine, a substance primarily associated with the metabolism of caffeine, exhibited significantly lower epileptogenesis levels during the acute and latent phases. Although its relationship with epileptogenesis has not been extensively studied, theobromine has been shown to modulate hippocampal neuron function and provide neuroprotection by regulating adenosine receptors in mice [67]. Previous studies on rat models of transient global cerebral ischemia have suggested its neuroprotective effects by reducing lipid peroxidation and increasing glutathione levels, potentially preserving glutamatergic and GABAergic signaling [68]. Furthermore, daily theobromine intake has been linked to improved cognitive performance [69]. Considering these aspects, particularly its adenosine receptor antagonism, the findings of this study propose theobromine as a potential prognostic biomarker of TLE. Although it is also recognized as a health supplement with potential in epileptogenesis treatment, further research is required to confirm its clinical applicability.

During epileptogenesis, most metabolites in urine show a decreasing trend compared with those in healthy controls, with only a few exhibiting notable increases. In the acute phase, dihydrothymine, TMA, and thymol stand out, whereas 1-MH is prominent in the latent phase. These phase-specific elevations provide valuable insights into the metabolic changes occurring during epileptogenesis.

Dihydrothymine, a thymine nucleotide degradation product, is significantly increased in urine during the acute phase of epileptogenesis, suggesting its potential as a biomarker. Although no direct association with epilepsy has been reported, dihydropyrimidinase deficiency presents symptoms ranging from seizures to intellectual and growth impairments [70,71,72,73,74,75,76,77]. Dihydropyrimidine may cause DNA damage because oxidative stress from SE and excessive ROS production can lead to DNA damage, lipid peroxidation, and mitochondrial dysfunction [78]. Basbaus et al. reported that dihydropyrimidine accumulation forms DNA–protein crosslinks (DPCs), disrupting replication and transcription [79]. Although the relationship between epilepsy and DNA damage has been studied, research on DPCs in epilepsy remains limited, warranting further investigation into dihydrothymine’s role and biomarker potential.

TMA, produced by gut microbiota from choline- or carnitine-rich foods, is absorbed in the gut, converted to trimethylamine-N-oxide (TMAO) in the liver via flavin monooxygenases (FMOs), and excreted by the kidneys [80]. In this study, increased TMA levels were observed in the urine of rats during the acute phase of epileptogenesis. While trimethylaminuria (TMAU) has been reported in two epilepsy cases, its direct relationship with epilepsy remains unclear [81,82]. Previous studies suggest that TMA may influence seizures by alkalizing cells, opening gap junctions, prolonging seizure durations, and increasing neuronal sensitivity [83]. Additionally, certain TMA-producing bacteria, such as *Klebsiella pneumoniae* and *Eggerthella lenta*, have been associated with epilepsy, with proposed mechanisms involving microglial inflammatory responses and potential biomarker roles in refractory epilepsy [84,85]. However, the causal link between TMA, gut microbiota alterations, and seizure susceptibility has not been firmly established. While TMA has been suggested to disrupt blood–brain barrier integrity, in contrast to the protective role of TMAO, the functional significance of these changes in epileptogenesis remains obscure and warrants further experimental validation [86]. The observed increase in TMA levels during the acute phase may reflect metabolic stress, gut microbiota fluctuations, or liver dysfunction rather than a direct mechanistic role in epileptogenesis. Future studies incorporating direct assessments of gut microbiota composition, metabolic flux analyses, and functional experiments targeting TMA-related pathways are necessary to determine the precise role of TMA in epilepsy pathophysiology.

Thymol, primarily derived from thyme (*Thymus vulgaris*) essential oil, exhibits antimicrobial, antioxidant, anti-inflammatory, and neuroprotective properties, making it a promising compound in traditional medicine and drug development [87,88]. Thymol interacts with GABA_A_ receptors, enhancing chloride ion influx to induce neuronal hyperpolarization, reducing excitability, and mitigating seizure activity [89]. Furthermore, it shows synergistic effects with anticonvulsants such as gidazepam, supporting its potential as an adjunct therapy for seizure disorders [90]. Beyond its GABAergic effects, thymol enhances endogenous antioxidants such as superoxide dismutase and glutathione, reducing oxidative damage and supporting brain health [91]. Elevated urinary thymol levels during the acute phase may result from seizure-induced metabolic changes, oxidative stress, inflammation, or increased neuroprotective usage. Investigating thymol’s role in epileptogenesis could help develop novel antiepileptogenic strategies.

In the latent phase of epileptogenesis, urinary analysis revealed a decrease in all metabolites, except for 1-MH, which exhibited a notable increase. Although postmortem studies have reported reduced 1-MH levels in epileptic brain tissue [44], its connection to epilepsy remains underexplored. 1-MH, a marker of skeletal muscle metabolism, is released during the breakdown of myofibrillar proteins such as actin and myosin [92,93,94,95]. Elevated 1-MH levels may reflect changes in muscle protein turnover due to seizure-related muscle activity or oxidative stress. As a byproduct of anserine, 1-MH is associated with histidine metabolism, which produces dipeptides such as carnosine with neuroprotective antioxidant properties [96]. Because oxidative stress plays a key role in epilepsy pathophysiology, studying 1-MH and related metabolites such as anserine and carnosine could enhance the understanding of latent-phase biomarkers and therapeutic targets. Further research into histidine metabolism may uncover new strategies for treating epilepsy.

This study has identified several notable metabolites that are present in the chronic phase of epileptogenesis. These include pimelic acid, tiglylglycine, D-lactose, and xanthurenic acid. Although their direct association with epilepsy remains unclear, their effects are exerted through various mechanisms. Pimelic acid, a seven-carbon dicarboxylic acid, is involved in biotin biosynthesis and fatty acid metabolism, potentially connecting it to mitochondrial function and oxidative stress in patients with epilepsy [97]. Tiglylglycine, a glycine derivative, accumulates under conditions of impaired isoleucine catabolism, often associated with enzyme deficiencies such as beta-ketothiolase, suggesting a potential association with mitochondrial dysfunction [98]. D-lactose, a sugar involved in carbohydrate metabolism, is essential for brain energy homeostasis, and disruptions in its metabolism may influence neuronal excitability [99]. Xanthurenic acid, a metabolite in the kynurenine pathway of tryptophan metabolism, is associated with excitotoxicity and seizure activity. Dysregulation in this pathway has been linked to epilepsy, and chronic levetiracetam use has been reported to increase kynurenic and xanthurenic acid levels [100]. These metabolites warrant further research to explore their potential as biomarkers and therapeutic targets for epilepsy.

While our study provides valuable insights into metabolic alterations during epileptogenesis using urinary metabolomics, certain limitations must be acknowledged. One primary constraint is the lack of direct correlation between urinary metabolic changes and brain tissue metabolism. Future studies incorporating plasma, serum, or CSF metabolomics alongside urinary profiling would strengthen the relevance of these findings to brain metabolism and systemic metabolic disturbances. Additionally, while our study identifies significant metabolic alterations, it does not assess their functional implications in terms of seizure susceptibility, neuronal excitability, or oxidative stress markers. To establish causality, future research should include functional validation experiments, such as enzyme activity assays, oxidative stress assessments, or targeted metabolic interventions in epilepsy models. Another important limitation is the use of only male rats, which restricts the generalizability of our findings. Future studies should include female animals to provide a more comprehensive understanding of metabolic changes across sexes. Moreover, our study focused on a six-week period, which may not fully capture the long-term trajectory of metabolic alterations during epilepsy progression. Extending the monitoring period in future research would help determine whether the identified biomarkers persist, fluctuate, or evolve over time. These efforts will enhance the translational potential of urinary metabolomics in epilepsy research and improve the identification of reliable metabolic biomarkers for disease progression and therapeutic response.

## 5. Conclusions

This study comprehensively analyzed the urinary metabolic profiles during the three main phases of epileptogenesis in a lithium–pilocarpine-induced TLE rat model, focusing on post-SE metabolic changes and associated pathways. The results revealed significant metabolite alterations and pathway disruptions, particularly in the acute phase of epileptogenesis. Notably, significant changes have also been observed in the latent period, although very few in the chronic period. It is noteworthy that the metabolites and metabolic pathways that were identified were consistently associated with energy metabolism across all the stages that were examined. The findings highlight the potential of urinary metabolites as biomarkers of epileptogenesis, thereby establishing a framework for elucidating SE-driven mechanisms and developing targeted therapeutic strategies. Future studies should focus on validating the urinary metabolites identified as reliable biomarkers for different stages of epileptogenesis. Moreover, investigating these metabolites and pathways in clinical settings and diverse epilepsy models may facilitate the development of targeted therapeutic strategies and novel interventions.

## Figures and Tables

**Table 1:** Metabolites showing ≥1.5-fold change in urine in the acute phase of epileptogenesis.

Metabolites	Fold Change (FC)	log2(FC)
Benzoic acid	43.705	5.4497
Trimethylamine	34.98	5.1285
Dihydrocuracil	31.3	4.9681
Thymol	20.513	4.3585
Dihydrothymine	17.792	4.1532
Citric acid	0.081913	−3.6
N,N-Dimethylglycine	0.086644	−3.5287
Orotic acid	0.088179	−3.5034
Allantoin	0.10589	−3.2393
Acetic acid	9.1855	3.1994
Uracil	0.13504	−2.8885
Fumaric acid	0.14091	−2.8272
DL-Alloisoleucine	6.9253	2.7919
Taurine	0.14904	−2.7463
3-Hydroxybutyric acid	6.2851	2.6519
2-Oxoglutaric acid	0.1685	−2.5692
Hippuric acid	0.18588	−2.4276
L-Pyroglutamic acid	0.19772	−2.3384
Glycolic acid	4.9651	2.3118
Cytosine	4.8923	2.2905
D-Galactonic acid	0.21173	−2.2397
Thymine	4.6909	2.2299
Adenine	0.22812	−2.1321
Trigonelline	0.22894	−2.1269
Tiglylglycine	0.23301	−2.1015
D-Glucose	0.23765	−2.0731
Neopterin	0.23989	−2.0596
Formic acid	3.7881	1.9215
Choline	3.7619	1.9115
2-Hydroxyphenylacetic acid	0.29104	−1.7807
Arginine	3.4255	1.7763
N-Acetylphenylalanine	3.3872	1.7601
2-Furoylglycine	0.30426	−1.7166
Tartaric acid	0.30429	−1.7165
Propionic acid	3.0572	1.6122
4-Hydroxyphenyllactic acid	3.0516	1.6096
L-Fucose	3.0012	1.5855
Theobromine	0.33673	−1.5703
Paracetamol	0.33909	−1.5602
1-Methylhydantoin	0.36512	−1.4535
Acetoacetic acid	2.6804	1.4224
N-Isovaleroylglycine	2.6404	1.4008
3-Phenyllactic acid	0.38041	−1.3944
3-Hydroxyglutaric acid	2.6254	1.3926
Betaine	0.39463	−1.3414
Methylmalonic acid	0.42302	−1.2412
Methionine	0.42857	−1.2224
2-Hydroxy-4-methylvaleric acid	0.42857	−1.2224
Butyric acid	0.42857	−1.2224
D-Gluconic acid	0.42857	−1.2224
4-Hydroxyphenylpyruvic acid	0.42857	−1.2224
Caffeine	2.2878	1.194
Methanol	2.2271	1.1552
Pyruvic acid	0.45075	−1.1496
Alanine	2.2094	1.1437
Phenylalanine	0.45539	−1.1348
D-Lactose	0.45752	−1.1281
Syringic acid	0.47699	−1.068
Phenylacetic acid	2.0575	1.0409
3-Methylglutaconic acid	2.0519	1.0369
1-Methylnicotinamide	0.4895	−1.0306
D-Galactose	2.0303	1.0217
L-Citramalic acid	1.9516	0.96463
Phenylpyruvic acid	1.8096	0.85567
1-Methylhistidine	1.8	0.848
2-Methylsuccinic acid	1.8	0.848
3-Hydroxyvaleric acid	1.8	0.848
Pimelic acid	1.8	0.848
3-Hydroxypropionic acid	1.8	0.848
Malic acid	1.8	0.848
1-Methyladenosine	1.8	0.848
D-Mannose	1.8	0.848
Myo-Inositol	1.8	0.848
Creatine	1.7929	0.84229
3-Dimethyluric acid	0.56122	−0.83336
Propylene glycol	0.5736	−0.80188
Glycine	1.7404	0.7994
Creatinine	1.7165	0.7795
Pantothenic acid	1.6383	0.71218
Oxypurinol	0.61067	−0.71154
L-Isoleucine	0.62857	−0.66985
4-Aminobutyric acid	1.5752	0.65556
3-Hydroxyisovaleric acid	0.64777	−0.62645
2-Hydroxyisovaleric acid	1.5368	0.61992

**Table 2:** Metabolites showing ≥1.5-FC in urine in the latent phase of epileptogenesis.

Metabolites	FC	log2(FC)
Tiglylglycine	0.048578	−4.3635
Benzoic acid	0.18757	−2.4145
3-Methylglutaconic acid	0.18868	−2.406
1-Methylhistidine	4.2611	2.0912
Neopterin	0.27284	−1.8739
3-Methyl−2-oxovaleric acid	0.27591	−1.8577
Phenylacetic acid	0.29675	−1.7527
Malic acid	2.9057	1.5389
Trimethylamine	2.8778	1.525
Dihydrothymine	0.37171	−1.4278
Thymol	0.37841	−1.402
D-Glucose	0.39546	−1.384
2-Hydroxyisovaleric acid	0.39612	−1.336
Theobromine	0.40343	−1.3096
D-Lactose	0.41697	−1.262
Glycine	0.41906	−1.2548
D-Galactose	0.42087	−1.2486
Proline betaine	0.44384	−1.1719
Uracil	0.46255	−1.1123
1-Methylnicotinamide	0.46326	−1.1101
4-Hydroxyphenylpyruvic acid	0.46667	−1.0995
Creatine	2.1418	1.0989
Valine	0.46809	−1.0952
D-Mannose	0.47201	−1.0831
Leucine	0.47377	−1.0778
1.3-Dimethyluric acid	0.47713	−1.0675
Taurine	0.47974	−1.0597
Thymine	0.48523	−1.0433
1-methylguanidine	0.4859	−1.0413
Hippuric acid	0.48611	−1.0407
Glycolic acid	0.49544	−1.0132
N-Acetylphenylalanine	0.49872	−1.0037
Arginine	0.50026	−0.99924
Pantothenic acid	0.51017	−0.97095
Guanidinoacetic acid	0.51046	−0.97013
N,N-Dimethylglycine	0.51694	−0.95195
Caffeine	0.51759	−0.95013
Allantoin	0.52538	−0.92857
Orotic acid	0.52896	−0.91877
1-Methylhydantoin	0.5327	−0.90861
Oxaloacetic acid	0.53417	−0.90463
2-Oxoglutaric acid	1.8689	0.90216
Paracetamol	0.54391	−0.87857
Creatinine	0.55068	−0.86072
Cytosine	1.8114	0.85711
Trigonelline	0.55488	−0.84974
Cystine	1.8	0.848
2-Methylsuccinic acid	1.8	0.848
D-Galactonic acid	1.8	0.848
2-Ketobutyric acid	1.8	0.848
Succinylacetone	1.8	0.848
Betaine	0.55906	−0.83894
N-Isovalerylglycine	0.56224	−0.83075
4-Pyridoxic acid	0.57043	−0.80988
3-Hydroxyisovaleric acid	0.57841	−0.78983
Oxypurinol	0.58576	−0.77162
Syringic acid	0.59278	−0.75442
L-Tryptophan	0.60433	−0.72659
3-Hydroxyglutaric acid	0.60896	−0.71558
Formic acid	1.608	0.6853
Phenylalanine	0.62224	−0.68446
L-Pyroglutamic acid	0.63154	−0.66304
2-Furoilglycine	0.63636	−0.65208
Methionine	0.63636	−0.65208
N-Acetylglutamate	0.63636	−0.65208
4-Hydroxyphenyllactic acid	0.63636	−0.65208
2-Hydroxy-4-methylvaleric acid	0.63636	−0.65208
3-Hydroxy-3-methylglutaric acid	0.63636	−0.65208
3-Hydroxypropionic acid	0.63636	−0.65208
2-Oxoisovaleric acid	0.63636	−0.65208
Dihydrouracil	0.63636	−0.65208
Myo-Inositol	0.63636	−0.65208
Uridine	0.63693	−0.65079
Alanine	0.64244	−0.63837
N-Acetylaspartic acid	0.64712	−0.62789
Inosine	0.65154	−0.61808
Sarcosine	0.65246	−0.61605
Xanthurenic acid	0.65455	−0.61143
2-Oxoisocaproic acid	0.65645	−0.60725
Tartaric acid	0.65763	−0.60466
Citric acid	0.65918	−0.60126
3-Hydroxybutyric acid	0.66605	−0.5863

**Table 3:** Metabolites showing ≥1.5-FC in urine in the chronic phase of epileptogenesis.

Metabolites	FC	log2(FC)
Pimelic acid	0.080808	−3.6294
1-Methylhistidine	7.3571	2.8791
Butyric acid	0.17885	−2.4832
Tiglylglycine	0.18451	−2.4382
D-Lactose	0.31879	−1.6493
4-Aminobutyric acid	2.851	1.5115
Imidazole	2.8099	1.4905
4-Hydroxyphenylpyruvic acid	0.35885	−1.4785
Creatine	0.37199	−1.4267
2-Hydroxyphenylacetic acid	0.38591	−1.3737
2-Ketobutyric acid	0.38777	−1.3667
Dihydrothymine	0.39207	−1.3508
D-Gluconic acid	2.5361	1.3426
Choline	0.405	−1.304
Sarcosine	2.4044	1.2657
Thymol	2.3835	1.2531
3-Phenyllactic acid	0.42552	−1.2327
Xanthurenic acid	0.44266	−1.1757
Ethylmalonic acid	0.51293	−0.96317
Thymine	0.51528	−0.95657
Citric acid	0.51923	−0.94556
Adenine	0.56046	−0.83531
2-Hydroxyisovaleric acid	0.56099	−0.83395
Phenylpyruvic acid	0.56852	−0.81473
Leucine	0.57092	−0.80864
3-Hydroxyglutaric acid	0.59316	−0.7535
N-Acetyl-L-phenylalanine	0.6	−0.73697
2-Oxizovaleric acid	0.6	−0.73697
1-Methylnicotinamide	16.443	0.71746
Glutaric acid	0.61133	−0.70997
Theobromine	0.61982	−0.69008
Lactic acid	1.5643	0.64554
Hippuric acid	0.65055	−0.62027
3-Methyl-2-oxovaleric acid	0.65899	−0.60168
Propionic acid	0.66312	−0.59265

**Table 4:** Urinary metabolites that vary statistically significantly during different periods of epileptogenesis.

Groups	Metabolites	FC	log2(FC)	*p*	−log(p)	Increased/Decreased
SE-48h vs. C-48h	Acetic acid	9.185	3.199	0.0003	3.456	Increased
	Orotic acid	0.088	−3.503	0.0005	3.3419	Decreased
	Thymol	2.051	4.358	0.0006	3.251	Increased
	N,N-Dimethylglycine	0.086	−3.528	0.0009	3.069	Decreased
	Dihydrothymine	1.779	4.153	0.001	2.869	Increased
	Pyruvic acid	0.450	−1.149	0.002	2.738	Decreased
	Theobromine	0.336	−1.570	0.003	2.493	Decreased
	Trimethylamine	3.498	5.128	0.004	2.397	Increased
	Tartaric acid	0.304	−1.716	0.012	1.936	Decreased
	Allantoin	0.105	−3.239	0.014	1.864	Decreased
	Fumaric acid	0.140	−2.827	0.014	1.854	Decreased
	Hippuric acid	0.185	−2.427	0.015	1.821	Decreased
	2-Oxoglutaric acid	0.168	−2.569	0.017	1.762	Decreased
	Citric acid	0.081	−3.609	0.018	1.753	Decreased
	Taurine	0.149	−2.746	0.022	1.659	Decreased
	L-Pyroglutamic acid	0.197	−2.338	0.025	1.594	Decreased
	Trigonelline	0.228	−2.126	0.027	1.575	Decreased
	1-Methylhydantoin	0.36	−1.453	0.0301	1.522	Decreased
	Adenine	0.228	−2.132	0.031	1.506	Decreased
	Tiglylglycine	0.233	−2.101	0.035	1.452	Decreased
SE-1wk vs. C-1wk	Neopterin	0.273	−1.874	0.004	2.438	Decreased
	D-Glucose	0.396	−1.338	0.009	2.045	Decreased
	3-Methylglutacononic acid	0.189	−2.406	0.010	1.996	Decreased
	Valine	0.468	−1.095	0.011	1.942	Decreased
	Glycolic acid	0.495	−1.013	0.013	1.891	Decreased
	Taurine	0.48	−1.06	0.014	1.857	Decreased
	Theobromine	0.403	−1.31	0.014	1.841	Decreased
	Caffeine	0.518	−0.95	0.015	1.826	Decreased
	Uracil	0.463	−1.112	0.015	1.825	Decreased
	1-Methylhistidine	4.261	2.091	0.015	1.819	Increased
	Allantoin	0.525	−0.929	0.015	1.814	Decreased
	Guanidinoasetic acid	0.51	−0.97	0.015	1.8124	Decreased
	Hippuric acid	0.486	−1.041	0.02	1.684	Decreased
	Creatinine	0.551	−0.861	0.022	1.644	Decreased
	Proline betaine	0.444	−1.172	0.028	1.557	Decreased
	1-Methylguanidine	0.486	−1.041	0.029	1.531	Decreased
	4-Pyridoxic acid	0.57	−0.81	0.03	1.518	Decreased
	Pantothenic acid	0.511	−0.971	0.031	1.505	Decreased
	Alanine	0.642	−0.64	0.039	1.414	Decreased
	Syringic acid	0.593	−0.754	0.04	1.393	Decreased
	Inosine	0.652	−0.618	0.043	1.365	Decreased
	1-Methylhydantoin	0.533	−0.91	0.048	1.322	Decreased
	Oxypurinol	0.586	−0.772	0.048	1.321	Decreased
SE-6wk vs. C-6wk	D-Lactose	0.319	−1.649	0.026	1.586	Decreased
	Tiglylglycine	0.185	−2.438	0.028	1.549	Decreased
	Pimelic acid	0.081	−3.629	0.034	1.463	Decreased
	Xanthurenic acid	0.443	−1.176	0.039	1.414	Decreased

**Table 5:** Urinary metabolites and metabolic pathways that change statistically significantly during different periods of epileptogenesis.

Metabolic Pathway (KEGG) (*Rattus norvegicus*)	SE-48h vs. C-48h		SE-1wk vs. C-1wk		SE-6wk vs. C-6wk	
	*p*	Metabolites	*p*	Metabolites	*p*	Metabolites
Alanine, aspartate, and glutamate metabolism	0.004	Citric acid Pyruvic acid Fumaric acid 2-Oxoglutaric acid				
Glyoxylate and dicarboxylate metabolism	0.002	Citric acid Pyruvic acid Acetic acid				
Glycine, serine, and threonine metabolism	0.014	N,N-Dimethylglycine Pyruvic acid				
TCA cycle (citrate cycle)	0.018	2-Oxoglutaric acid Citric acid Pyruvic acid Fumaric acid				
Pyruvate metabolism	<0.001	Pyruvic acid Fumaric acid Acetic acid				
Glycolysis/gluco-neogenesis	0.012	Pyruvic acid Acetic acid	0.006	D-Glucose		
Pyrimidine metabolism	0.011	Orotic acid				
Pentose phosphate pathway			0.009	D-Glucose		
Caffeine metabolism	0.003	Theobromine	0.012	Theobromine Caffeine		
Taurine and hypotaurine metabolism	0.022	Taurine	0.014	Taurine		
Purine metabolism	0.017	Allantoin	0.023	Allantoin Inosine		
Selenocomponent metabolism			0.039	Alanine		
Primary bile acid biosynthesis			0.031	Taurine		

## Data Availability

The raw data supporting the conclusions of this article will be made available by the corresponding author via email.

## Supplementary Materials

The following supporting information can be downloaded at: https://www.mdpi.com/article/10.3390/biomedicines13030588/s1, Figure S1. OPLS-DA permutation test validation for different phases of epileptogenesis. Permutation tests were performed to assess the robustness of the OPLS-DA models in distinguishing metabolic profiles between groups. (a) Acute phase shows strong predictive power (Q^2^ = 0.843, *p* = 0.01). (b) Latent phase exhibits moderate predictive ability (Q^2^ = 0.499, *p* = 0.06). (c) Chronic phase does not show significant predictive power (Q^2^ = −0.0335, *p* = 0.36).
