# Supplementary material for: Urinary Metabolic Profiling During Epileptogenesis in Rat Model of Lithium–Pilocarpine-Induced Temporal Lobe Epilepsy
Source: Biomedicines. 2025 Feb 27;13(3):588. doi: 10.3390/biomedicines13030588 (PMC11940187; doi:10.3390/biomedicines13030588)
Supplement: Supplementary file 1 [file biomedicines-13-00588-s001.zip › biomedicines-3440808-supplementary.pdf]

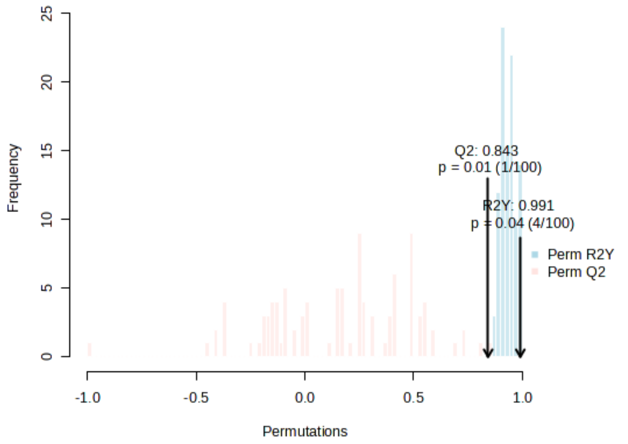

(**a**)

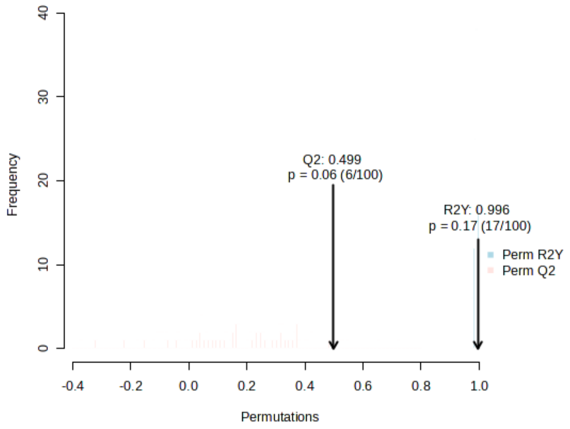

(**b**)

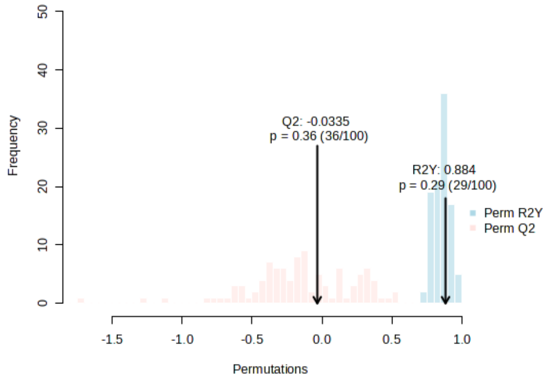

(**c**)

Figure S1. OPLS-DA permutation test validation for different phases of epileptogenesis. Permutation tests were performed to assess the robustness of the OPLS-DA models in distinguishing metabolic profiles between groups. (a) Acute phase shows strong predictive power (Q^2 = 0.843, p = 0.01). (b) Latent phase exhibits moderate predictive ability (Q^2 = 0.499, p = 0.06). (c) Chronic phase does not show significant predictive power (Q^2 = -0.0335, p = 0.36).
